# Nomogram for the prediction of surgical site infection following spinal surgery: a multicenter retrospective study

**DOI:** 10.3389/fmed.2026.1832891

**Published:** 2026-04-28

**Authors:** Yang Sun, Qi Li, Pengfei Zhai, Rui Zhang, Hongguang Wang, Xiaoguang Tong

**Affiliations:** 1 Huanhu Hospital Affiliated to Tianjin Medical University, Tianjin, China; 2 Huanhu Hospital, Tianjin University, Tianjin, China; 3 Tianjin Key Laboratory of Cerebral Blood Flow Reconstruction and Head and Neck Tumor New Technology Translation, Tianjin, China; 4 Department of Spinal Surgery, Honghui Hospital, Xi'an Jiaotong University, Xi’an, China

**Keywords:** nomogram, prediction model, risk factors, spinal surgery, surgical site infection

## Abstract

**Objective:**

Surgical site infection (SSI) represents a prevalent postoperative complication associated with spinal surgery, contributing to increased morbidity and mortality rates. This study sought to identify key prognostic factors for SSI following spinal surgery and to develop a novel nomogram to predict SSI incidence.

**Methods:**

Retrospective data collection was conducted on patients who underwent spinal surgeries between 2017 and 2024. The cohort was stratified into two groups: those with infections (*n* = 59) and those without infections (*n* = 990). A nomogram was developed to predict the risk of SSI outcomes, utilizing results derived from univariate and multivariate regression analyses of factors influencing SSI after spinal surgery. Internal validation of the nomogram was conducted through Bootstrap analysis.

**Results:**

A total of 1,049 patients were enrolled in the study. Variables identified as statistically significant through univariate regression analyses were incorporated into the multivariate regression model. The analysis revealed that age (odds ratio [OR]: 3.312, 95% confidence interval [CI]: 1.377–7.965), diabetes mellitus (OR: 3.698, 95% CI: 1.854–7.377), albumin levels (OR: 0.172, 95% CI: 0.091–0.326), operative time (OR: 2.003, 95% CI: 1.129–3.554), method of suture (OR: 0.459, 95% CI: 0.258–0.817), and blood loss (OR: 2.085, 95% CI: 1.183–3.674) were independent predictors. Based on these indicators, a nomogram model was developed. Routine bacterial cultures of surgical site secretions were performed in patients with suspected infections, revealing that *Staphylococcus aureus* was the most prevalent microorganism. The application of the nomogram in the validation cohort exhibited good discrimination ability, with a concordance index of 0.787 (95% CI, 0.718–0.856), and demonstrated good calibration. Decision curve analysis further confirmed the model’s superior clinical utility across a wide range of threshold probabilities.

**Conclusion:**

This study has developed a robust and valuable nomogram capable of accurately predicting the incidence of SSI following spinal surgery in patients. This tool is user-friendly and has the potential to aid clinicians in making informed clinical decisions tailored to individual patients.

## 1 Introduction

Surgical site infection (SSI) is a prevalent postoperative complication associated with spine surgery (1). The occurrence of postoperative incision infections contributes to increased morbidity and mortality rates and imposes a substantial economic burden on both patients and the healthcare system (2). Despite advancements in surgical instruments and techniques that have enhanced the capacity to address a wide array of complex spinal conditions, the incidence of postoperative incision infections has not shown a corresponding decline (3, 4). The intricate nature of spinal surgical procedures contributes to a higher SSI rate compared to other orthopedic surgeries (1, 5). The reported incidence of SSI following spinal surgery varies between 0.7 and 12%, reflecting differences in surgical approaches and technical complexity (1, 6). Previous research has identified several factors associated with SSI following surgery, including patient age, obesity, duration of surgery, blood loss, and the specific surgical segments involved (1, 3, 7, 8). However, there is a paucity of large-scale clinical studies addressing this issue (9). The risk factors examined in existing studies are not sufficiently comprehensive, and there is a lack of analysis regarding the correlations and significance of these risk factors. Furthermore, no prior studies have investigated the relationship between the method of incision suturing and the incidence of postoperative SSI. To the best of our knowledge, there is currently no established method for accurately predicting the probability of SSI.

In the current study, we conducted an extensive analysis of potential risk factors associated with SSI following spinal surgery. Utilizing these identified risk factors, our objective is to develop a robust and valuable nomogram to predict the likelihood of SSI after spinal surgery. To the best of our knowledge, no existing nomogram specifically addresses the prediction of SSI probability following such surgical procedures.

## 2 Materials and methods

### 2.1 Patients

This study protocol was reviewed and approved by the ethics committees of Tianjin Huanhu Hospital and Xi’an Honghui Hospital. The study was conducted in accordance with the latest revision of Declaration of Helsinki. Written and oral informed consent was obtained from patients.

**Figure 1:**
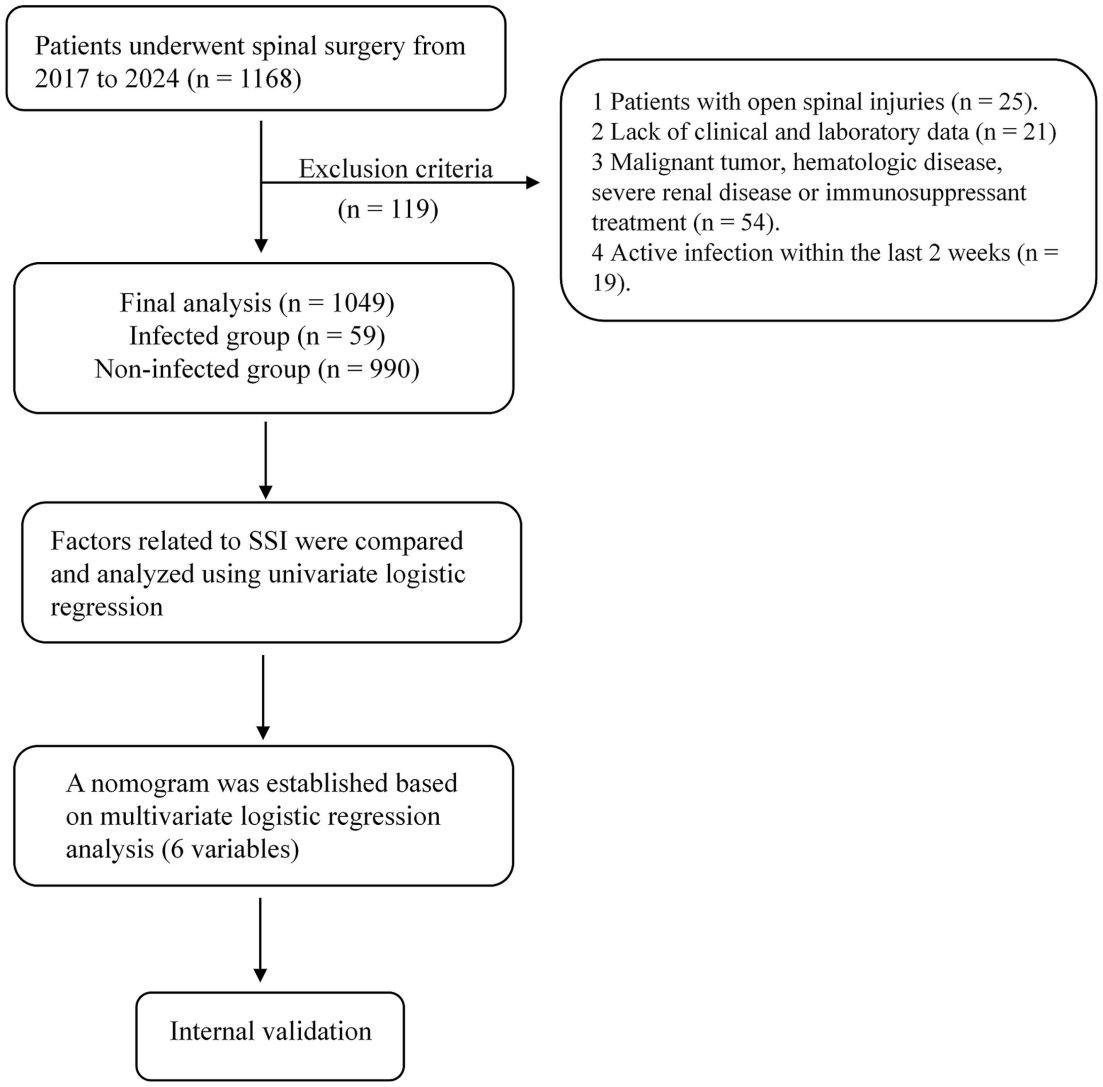
Workflow of the study.

A total of 1,049 consecutive patients who underwent spinal surgery at the Spine Surgery Departments of Tianjin Huanhu Hospital and Xi’an Honghui Hospital between January 1, 2017, and January 1, 2024, were retrospectively included in this study (Supplementary Table S1). The patients included in this study underwent spinal surgeries, specifically decompression or fusion. Surgical site infections (SSIs) were diagnosed based on the criteria established by the Centers for Disease Control and Prevention’s National Healthcare Safety Network (CDC/NHSN), including both superficial and deep incisional infections. The follow-up period was at least 30 days postoperatively, and up to 90 days for patients with spinal instrumentation (10). The cohort was subsequently divided into infected (*n* = 59) and non-infected (*n* = 990) groups. Exclusion criteria encompassed individuals with spinal infections or tuberculosis, spinal metastatic tumors, open spinal injuries, and those with significant immunodeficiency prior to surgery or long-term use of immunosuppressants (Figure 1).

### 2.2 Clinical examination and treatment

All procedures in this study were conducted by a consistent team of surgeons. Each patient was administered a standard regimen of systemic antibiotic prophylaxis, consisting of 1.5 g of cefuroxime. The first dose was administered intravenously 30–60 min before skin incision, and postoperative continuation followed our institutional infection control protocol, for a total of 3 days. Routine assessments comprised pre- and post-operative spinal radiography, pre- and post-operative spinal computed tomography (CT) scans, and preoperative spinal magnetic resonance imaging (MRI). For patients with suspected postoperative infections, bacterial cultures were performed.

### 2.3 Clinical variables

The clinical characteristics of the study participants are detailed in Table 1. These data were primarily extracted from the patients’ clinical medical records. Our analysis incorporated the following sociodemographic, clinical and laboratory variables: age, sex, body mass index (BMI), comorbidities (including cardiac disease, hypertension, and diabetes mellitus), smoking and/or alcohol consumption within the past 12 months, American Society of Anesthesiologists (ASA) physical status classification score, operative duration, blood loss, surgical site (categorized as cervical, thoracic, or lumbar), surgical segment (classified into groups of fewer than 2 segments and more than 2 segments), surgical approach (anterior, posterior, or combined anterior and posterior approaches), suture technique (continuous intradermal running suture versus simple interrupted percutaneous suture), and hematological indices such as serum total protein, globulin, albumin, leukocyte count, lymphocyte count, and neutrophil count.

**Table 1:** Baseline characteristics between infected group and non-infected group.

Characteristics	Non-infected group (*N* = 990)	Infected group (*N* = 59)	*p* Value^a^
Age (years)	51.17 ± 15.67	56.03 ± 13.08	0.020*
Gender (*n*, %)			0.718
Female	446 (45.1%)	28 (47.5%)	
Male	544 (54.9%)	31 (52.5%)	
BMI (kg/m^2^)	24.99 ± 10.05	25.14 ± 9.97	0.914
Smoking (*n*, %)			0.635
No	590 (59.6%)	37 (62.7%)	
Yes	400 (40.4%)	22 (37.3%)	
Alcohol use (*n*, %)			0.859
No	694 (70.1%)	42 (71.2%)	
Yes	296 (29.9%)	17 (28.8%)	
Cardiac disease (*n*, %)			0.428
No	888 (89.7%)	51 (86.4%)	
Yes	102 (10.3%)	8 (13.6%)	
Hypertension (*n*, %)			0.864
No	498 (50.3%)	29 (49.2%)	
Yes	492 (49.7%)	30 (50.8%)	
Diabetes mellitus (*n*, %)			0.002*
No	887 (89.6%)	45 (76.3%)	
Yes	103 (10.4%)	14 (23.7%)	
Serum total protein (g/L)	62.88 ± 10.05	63.05 ± 10.87	0.897
Globulin (g/L)	24.53 ± 4.39	25.05 ± 4.44	0.376
Albumin (g/L)	42.36 ± 5.96	38.24 ± 5.00	<0.001*
Leukocyte (10^9^/L)	7.55 ± 0.83	7.58 ± 0.83	0.789
Lymphocyte (10^9^/L)	1.55 ± 0.50	1.50 ± 0.46	0.444
Neutrophil (10^9^/L)	4.34 ± 0.59	4.42 ± 0.61	0.350
ASA score	1.94 ± 0.57	2.00 ± 0.56	0.403
Operative time (min)	182.19 ± 50.55	201.19 ± 52.69	0.005*
Blood loss (ml)	439.70 ± 206.54	494.92 ± 207.55	0.046*
Surgical site			0.069
Cervical	248 (25.1%)	9 (15.2%)	
Thoracic	171 (17.2%)	7 (11.9%)	
Lumbar	571 (57.7%)	43 (72.9%)	
Surgical segment			0.338
<2 spinal segments	415 (41.9%)	21 (35.6%)	
≥2 spinal segments	575 (58.1%)	38 (64.4%)	
Surgical approach			0.084
Anterior	139 (14.0%)	13 (22.0%)	
Posterior	777 (78.5%)	39 (66.1%)	
A and P combination	74 (7.5%)	7 (11.9%)	
Method of suture			0.042*
Intradermal suture	487 (49.2%)	21 (35.6%)	
Interrupted suture	503 (50.8%)	38 (64.4%)	

^a^*χ*^2^ and Fisher’s exact test were used for categorical variables, and Student’s *t* test was used for continuous variables. *Statistically significant. BMI, Body mass index; ASA, American Society of Anesthesiologists; A, Anterior; P, Posterior.

### 2.4 Statistical analysis

Statistical analyses were conducted utilizing SPSS software, version 22.0, for Windows (IBM Corp., Armonk, NY, USA). Quantitative variables were examined for normal distribution using Shapiro–Wilk test and were presented as mean ± standard deviation for normally distributed variables and median with interquartile range for non-normally distributed variables. Single-factor analyses were performed using the *t*-test, Chi-square (*χ*^2^) test, or Fisher’s exact test. The significance of each variable was evaluated through univariate logistic regression analysis. Subsequently, variables identified as significantly associated with SSI were incorporated into the multivariate model. Receiver operating characteristic (ROC) curve analysis was used to determine the optimal cutoff value for the continuous variable. The determination of optimal cutoff values for continuous variables was achieved through the application of the Youden index derived from ROC curve analysis. A *p*-value of less than 0.05 was considered indicative of statistical significance in all analyses. All variables included in the analysis had complete data, as they are routinely documented in the perioperative records.

A nomogram was constructed using the ‘rms’ package in R, version 3.0,^1^ based on the multivariate logistic regression analysis results (11). To validate the model, a widely accepted internal validation approach was employed. First, model discrimination was assessed using the concordance index (C-index), measured by the area under the ROC curve. A value of 1 signifies perfect discrimination, while a value of 0.5 indicates no discriminatory power. Second, model calibration was validated using a calibration curve, a graphical method in which a slope of 1 for the calibration curve at each point indicates optimal calibration. Goodness-of-fit was assessed using the Hosmer–Lemeshow test, with *p* > 0.05 indicating satisfactory model fit. Finally, decision curve analysis (DCA) was performed to evaluate the clinical utility of the model by quantifying the net benefit at different threshold probabilities.

## 3 Results

### 3.1 Patient characteristics

A total of 1,049 patients were ultimately included in the study. Table 1 presents a comparison of the characteristics between the infected and non-infected groups. Patients in the infected group were older and exhibited lower albumin levels compared to those in the non-infected group. Additionally, the infected group experienced longer operative time and greater intraoperative blood loss. There was also a higher incidence of diabetes among patients in the infected group. Furthermore, the method of surgical suturing may influence the incidence of postoperative SSIs.

### 3.2 Univariate analysis of variables in infected/non-infected group

Through univariate logistic regression analysis shown in Table 2, albumin levels in infected group were significantly lower than those in non-infected group (*p* < 0.001). Older (OR: 0.271, 95% CI: 0.115–0.636) and diabetic patients (OR: 0.373, 95% CI: 0.198–0.703) were correlated with SSI. The infected group had a significantly longer operation time (*p* = 0.009) and greater blood loss (*p* = 0.026) compared to the non-infected group. We also found that the method of suture was significantly associated with SSI (*p* = 0.045). In contrast, SSI was not related to other variables (*p* > 0.05). It is important to highlight that the *p*-value for the surgical site in the univariate logistic regression analysis was approximately 0.05 (*p* > 0.05). Given the critical significance of the surgical site in clinical practice, this variable was also included in the multivariate regression analyses.

**Table 2:** Univariate regression analysis of surgical site infection after spinal surgery.

Characteristics	OR (95% CI)	*p* Value
Age (years)		0.003*
<39.5	1	
>39.5	0.271 (0.115–0.636)	
Gender (*n*, %)		0.718
Female	1	
Male	1.102 (0.651–1.864)	
BMI (kg/m^2^)	1.001 (0.976–1.028)	0.914
Smoking (*n*, %)		0.636
No	1	
Yes	1.140 (0.663–1.962)	
Alcohol use (*n*, %)		0.860
No	1	
Yes	1.054 (0.590–1.881)	
Cardiac disease (*n*, %)		0.429
No	1	
Yes	0.732 (0.338–1.586)	
Hypertension (*n*, %)		0.864
No	1	
Yes	0.955 (0.565–1.615)	
Diabetes mellitus (*n*, %)		0.002*
No	1	
Yes	0.373 (0.198–0.703)	
Serum total protein (g/L)	1.002 (0.976–1.028)	0.897
Globulin (g/L)	1.027 (0.968–1.091)	0.376
Albumin (g/L)		<0.001*
≤41.5	1	
>41.5	4.987 (2.701–9.208)	
Leukocyte (10^9^/L)	1.044 (0.762–1.431)	0.789
Lymphocyte (10^9^/L)	0.813 (0.479–1.380)	0.444
Neutrophil (10^9^/L)	1.237 (0.792–1.932)	0.350
ASA score	1.218 (0.768–1.930)	0.402
Operative time (min)		0.009*
<180	1	
≥180	0.487 (0.283–0.838)	
Blood loss (ml)		0.026*
<475	1	
>475	0.548 (0.324–0.930)	
Surgical site		
Cervical	1	0.076
Thoracic	0.482 (0.231–1.004)	0.051
Lumbar	0.544 (0.240–1.230)	0.144
Surgical segment		0.339
<2 spinal segments	1	
≥2 spinal segments	0.766 (0.443–1.324)	
Surgical approach		
Anterior	1	0.090
Posterior	0.537 (0.279–1.031)	0.062
A and P combination	1.011 (0.387–2.645)	0.982
Method of suture		0.045*
Intradermal suture	1	
Interrupted suture	0.571 (0.330–0.987)	

BMI, Body mass index; ASA, American Society of Anesthesiologists; A, Anterior; P, Posterior; OR, odds ratio; CI, confidence interval. *Statistically significant.

### 3.3 Correlation analysis and screening of variables

**Figure 2:**
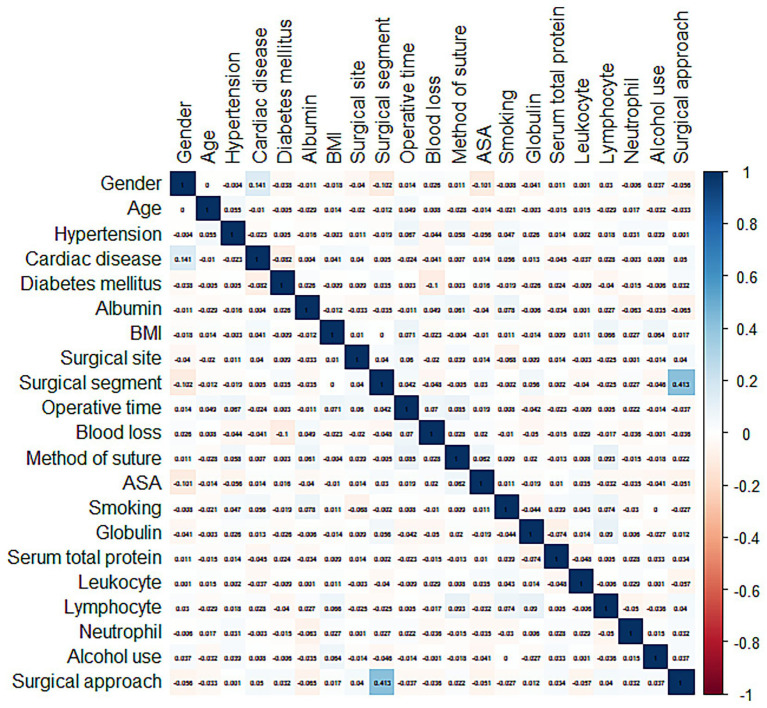
Correlation between variables.

Prior to conducting the multivariate logistic regression analysis, Spearman’s correlation test was employed to assess the collinearity among the risk factors. The resulting heat map showed that the highest correlation coefficient among the variables was 0.413, indicating no severe multicollinearity. Therefore, all seven variables were included in the subsequent multivariate analysis (Figure 2).

Based on the ROC curves for survival prediction, the optimal cut-off values for age (AUC: 0.597), albumin (AUC: 0.685), operative time (AUC: 0.588), and blood loss (AUC: 0.574) were identified as 39.5, 41.5, 180, and 475, respectively (Table 2; Supplementary Figures S1–S4). Subsequently, patients were categorized into high and low groups according to the respective cut-off values of these variables.

### 3.4 Multivariate logistic regression analysis of SSI

Multivariate logistic regression with significant variable in univariate analysis was employed to analyze the independent predictors of SSI. The factors analyzed included age, diabetes mellitus, albumin levels, operative time, suture method, blood loss, and surgical site. Categorical variables were classified using a binary assignment system, where a value of 0 represented “no” or “weak,” and a value of 1 represented “yes” or “strong.” The surgical site variable was categorized as follows: cervical (0), thoracic (1), and lumbar (2). The analysis employed a stepwise method for variable selection in the multivariate logistic regression, with a model inclusion threshold of 0.05 and a rejection threshold of 0.10. The results of the likelihood ratio chi-squared test indicated that the regression model was statistically significant (*p* < 0.05). Furthermore, the Hosmer–Lemeshow goodness-of-fit test demonstrated that the model exhibited an excellent degree of calibration (χ^2^ = 5.534; *p* = 0.699). As shown in Table 3, age, diabetes mellitus, albumin levels, operative time, suture method, and blood loss were identified as independent related factors of SSI, while the surgical site was not an independent related factor for SSI.

**Table 3:** Multivariate regression model of surgical site infection.

Variable	Wald	*p* value	OR	95% CI
Age	7.156	0.007	3.312	1.377–7.965
Diabetes mellitus	13.733	<0.001	3.698	1.854–7.377
Albumin	29.097	<0.001	0.172	0.091–0.326
Operative time	5.645	0.018	2.003	1.129–3.554
Method of suture	7.019	0.008	0.459	0.258–0.817
Blood loss	6.457	0.011	2.085	1.183–3.674
Surgical site				
Cervical	8.277	0.016	Ref	Ref
Thoracic	6.206	0.013	0.412	0.205–0.828
Lumbar	0.001	0.981	0.987	0.351–2.779

OR, odds ratio; CI, confidence interval; Ref, Reference.

### 3.5 Analysis of screening variables

**Figure 3:**
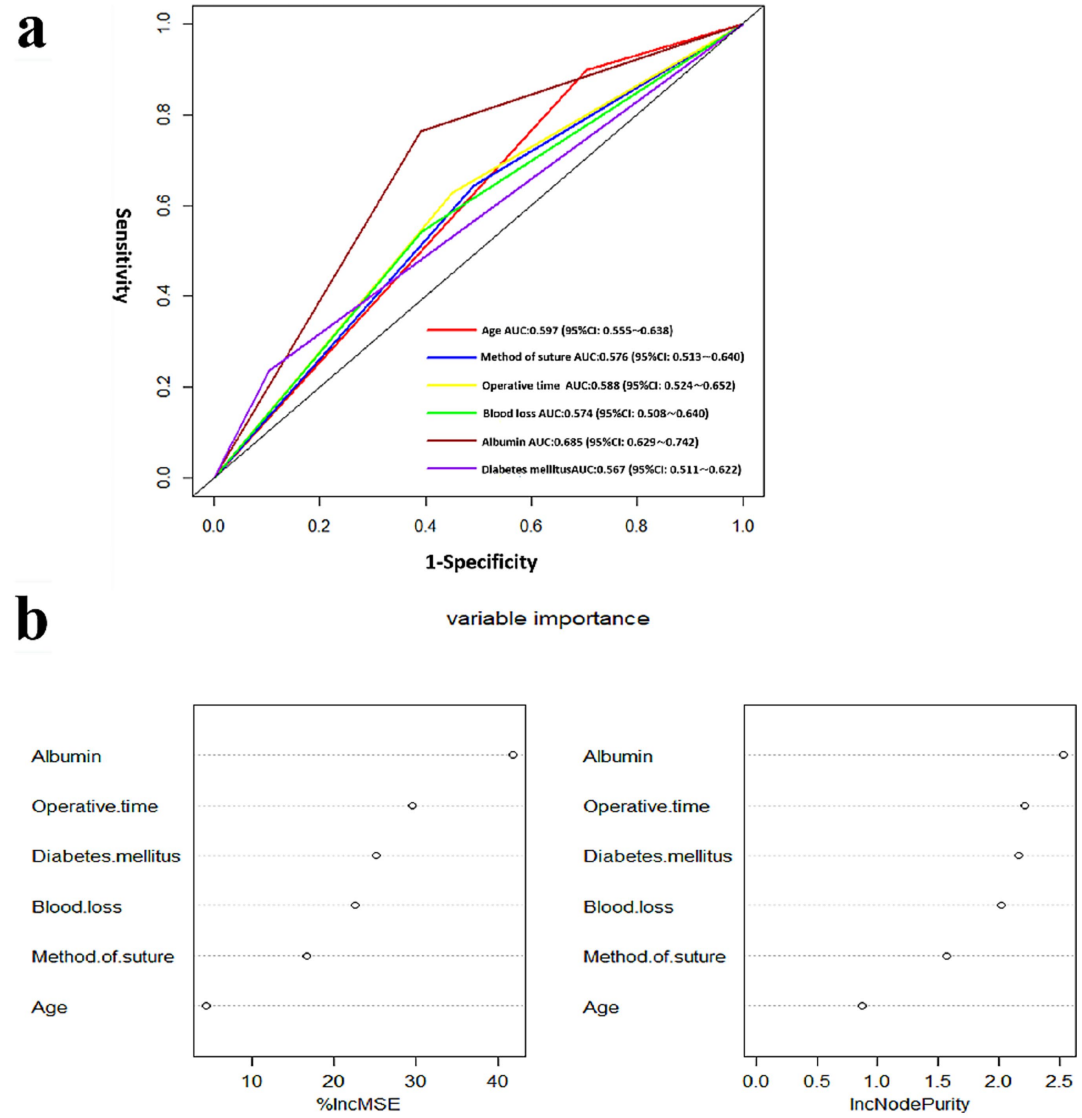
Diagnostic value and importance of variables: **(a)** Assessment of variables’ diagnostic value and **(b)** examination of variables’ importance.

The ROC curve was constructed based on screening variables for postoperative spinal infection identified through regression analysis, in order to evaluate the independent predictive value of these variables for SSI. As depicted in Figure 3a, the variable with the highest diagnostic value, as indicated by the AUC, is albumin. The AUCs for the aforementioned variables exceed 0.5, suggesting that their potential clinical utility in predicting SSI.

To identify all pertinent classified features based on their importance, six screening variables were analyzed. The importance of the independent variables was assessed using a random forest model (Figure 3b). Among the six factors, albumin emerged as the most important, followed by operative time and diabetes mellitus.

### 3.6 Nomogram of SSI after spinal surgery

**Figure 4:**
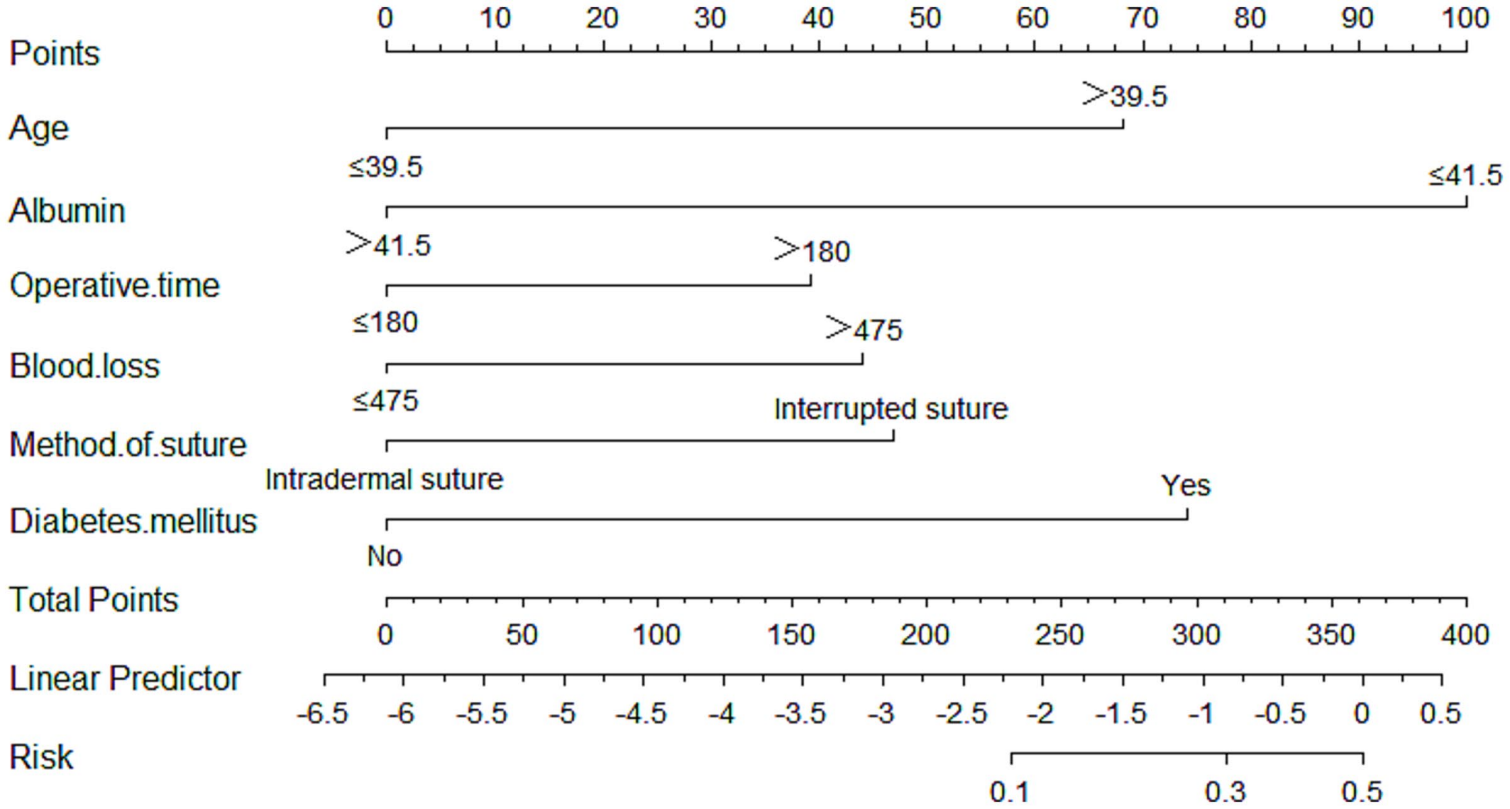
Nomogram for predicting the surgical site infection (SSI) following spinal surgery.

The nomogram for predicting SSI is presented in Figure 4. This nomogram was developed utilizing six independent predictive factors: age, diabetes mellitus, albumin level, operative time, suture method, and blood loss. Each factor within the nomogram is assigned a specific weighted point value, and the cumulative score for each patient corresponds to a particular SSI risk prediction. For instance, consider a 50-year-old patient (67.5 points) without diabetes mellitus (0 points), an albumin level of 30 g/L (100 points), an operative time of 200 min (40 points), intraoperative blood loss of 500 mL (45 points), and a simple interrupted percutaneous suture (47.5 points). The total score for this patient would be 300 points, which correlates to a 28% probability of developing a SSI following spinal surgery, as illustrated in Figure 4.

**Figure 5:**
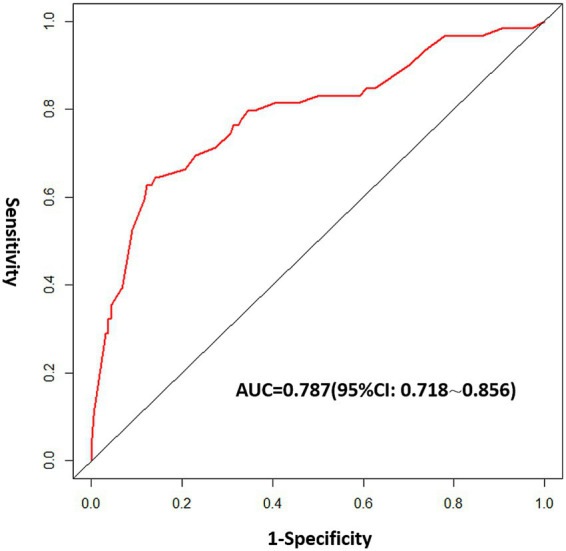
Receiver operating characteristic (ROC) curve analysis of the nomogram.

**Figure 6:**
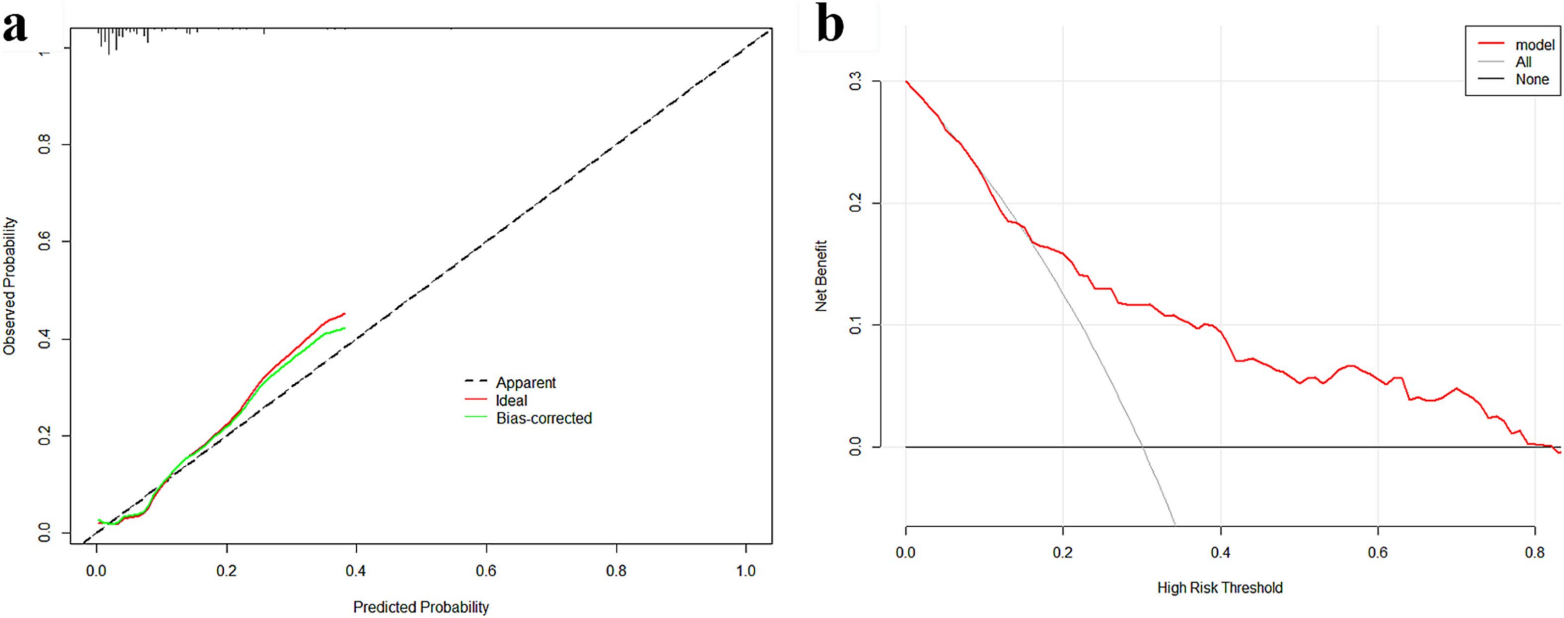
Validation of the nomogram: **(a)** Calibration curves of the nomogram. **(b)** Decision curve analysis (DCA) of the nomogram.

Internal validation of the nomogram was conducted through Bootstrap analysis. The C-index of the model was 0.787 (95% CI, 0.718–0.856), indicating that the model has good accuracy. The analysis results indicated that the area under the receiver operating characteristic curve (AUC_ROC_) was 0.787 (Figure 5), indicating that the model has good discrimination ability. The Hosmer–Lemeshow goodness-of-fit test was applied to assess the degree of agreement between the model’s predicted probability of the outcome event and the actual observed probability of the outcome event in real-world scenarios. In our study, the Hosmer–Lemeshow test produced a non-significant result (*p* = 0.699 > 0.05), suggesting adequate model fit. A calibration curve was generated by plotting the model’s predicted probability of the outcome event on the x-axis against the observed probability of the actual outcome event on the y-axis. The calibration curve serves as the most intuitive graphical representation of the Hosmer–Lemeshow goodness-of-fit test results. As shown in Figure 6a, the calibration curve demonstrated a high level of agreement between the model’s predicted probabilities and the observed outcomes, indicating that the model possesses good predictive performance. The DCA demonstrated that the model provided a higher net benefit compared with both the ‘treat-all’ and ‘treat-none’ strategies across the vast majority of threshold probabilities, confirming its robust clinical utility (Figure 6b). This nomogram is capable of predicting the risk of SSI on an individual basis, taking into account the unique conditions of different patients.

### 3.7 Etiology of SSI

**Figure 7:**
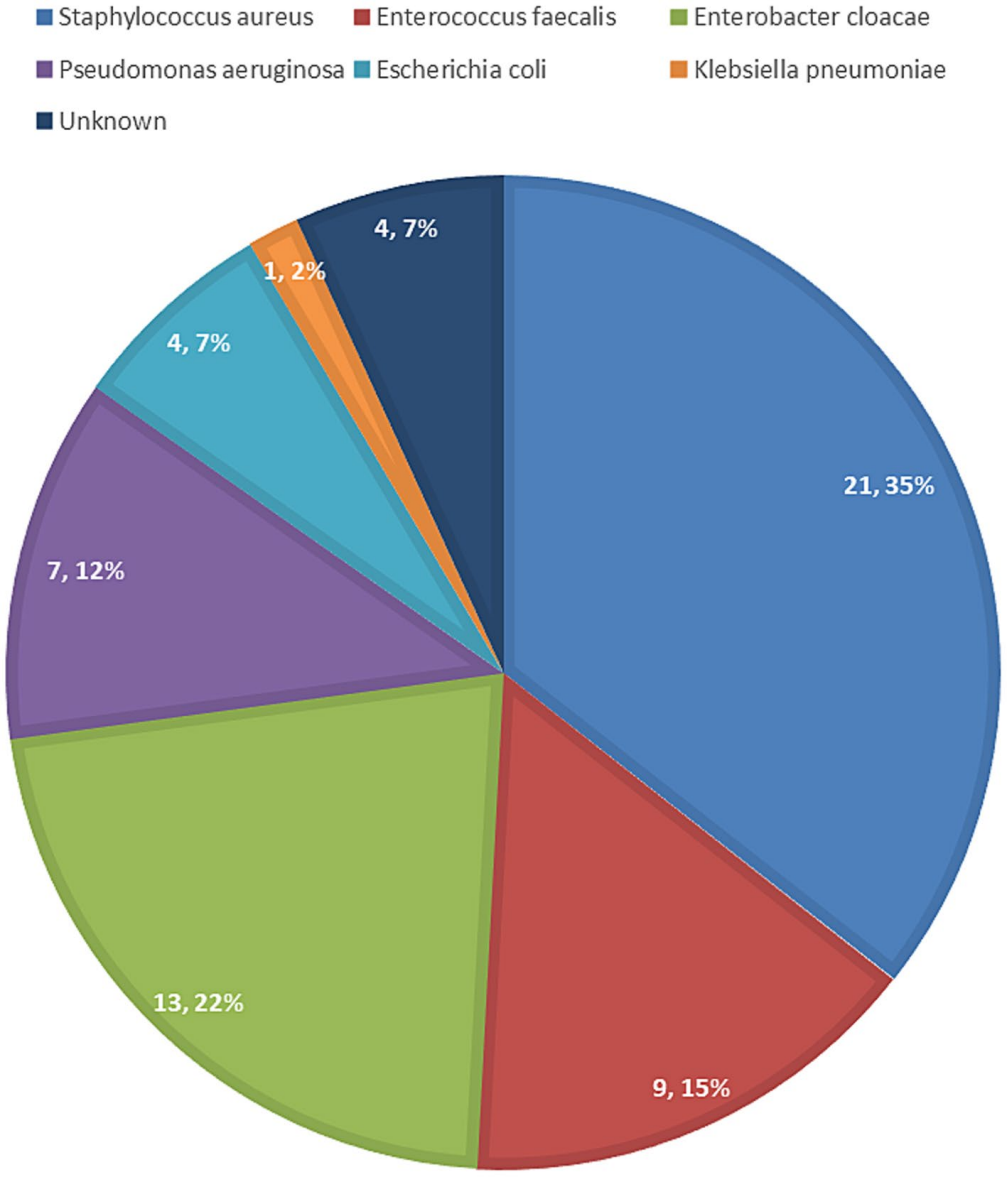
A fan shaped statistical chart of the microbiological characteristics of surgical site infection (SSI).

Patients with suspected infections typically undergo bacterial culture analysis of surgical site secretions. Our initial choice of cefuroxime sodium was informed by prior clinical experience in managing SSIs. Upon receiving the antimicrobial susceptibility test results, we tailored the antibiotic therapy accordingly. The predominant microorganisms identified in the bacterial cultures included *Staphylococcus aureus*, *Enterobacter cloacae*, and *Enterococcus faecalis* (Figure 7).

## 4 Discussion

Postoperative SSI represents a significant clinical challenge for spine surgeons and has considerable economic implications at the national level (12, 13). The financial burden of SSI ranges from $16,000 to $18,000 per case, resulting in total hospitalization costs exceeding 200% of the initial surgical expenses (14). Current literature reports postoperative spinal surgery infection rates between 1 and 10% (3, 4, 8, 13, 15), with variations attributable to the type of procedure, spinal segment involved, underlying pathology, and patient demographics. In the present study, approximately 5.6% of patients undergoing spinal surgery experienced incisional infections. Our findings indicate that factors such as age, diabetes mellitus, serum albumin levels, operative duration, suture technique, and intraoperative blood loss are significantly correlated with the incidence of postoperative SSI in a Chinese cohort. Given the detrimental impact of SSI on both patients and society, it is imperative for surgeons to conduct comprehensive investigations into the factors contributing to the development of SSI (5, 16, 17).

Few previous studies have developed nomograms for predicting SSI following spinal surgery. Namba et al. reported a prediction tool for high-risk SSI in spinal surgery with a C-index of 0.733 (18). Our nomogram achieved a C-index of 0.787 (95% CI: 0.718–0.856), suggesting comparable or slightly improved discrimination. Several other studies have focused primarily on the risk factors for SSI without developing a quantitative predictive model (1, 7, 19). The relatively higher performance of our model may be attributed to the inclusion of novel predictors such as suture method and albumin, as well as the multicenter design. However, direct comparisons should be interpreted with caution due to differences in study populations, outcome definitions, and follow-up durations.

Recent studies have identified several risk factors associated with postoperative spinal SSIs. In our research, we evaluated the significance and predictive value of these identified risk factors. Among them, albumin emerged as the most significant risk factor, both in terms of predictive value and importance. Serum albumin levels are indicative of a patient’s nutritional status, with lower levels suggesting malnutrition (8, 20, 21). A systematic review published in 2019 corroborated our findings, identifying malnutrition as a significant risk factor for the development of SSI following spinal surgery (14). Furthermore, hypoalbuminemia can compromise immune function and impair wound healing, thereby elevating the risk of SSI (8, 18). Our study identified reduced serum albumin levels as an independent risk factor for postoperative spinal infections. We further explored its critical threshold. As illustrated in Supplementary Figure S2, the critical serum albumin level for SSI was determined to be 41.5 g/L. The incidence of infection among patients with serum albumin levels ≤41.5 g/L was 9.2% (45 out of 433 patients). Consequently, it is imperative to prioritize the prevention of postoperative incisional infections in patients presenting with preoperative serum albumin levels below 41.5 g/L.

Age and diabetes mellitus were identified as independent risk factors for the development of postoperative incisional infections. In individuals with diabetes, the surgical incision is exposed to a highly oxidizing environment, which is linked to hyperglycemia and tissue hypoxia, subsequently delaying wound healing (22). Additionally, the compromised immune function observed in diabetic patients further elevates the risk of incisional infections (23). Kaye reported that age was a significant predictor of SSIs; however, the relationship between age and SSI risk was multifaceted (24). This complexity may be attributed to the presence of multiple comorbidities and malnutrition in elderly patients, which collectively impair their ability to effectively respond to infections.

Intraoperative factors are critically influential in elevating the risk of SSIs. Kurmann et al. reported that an operative duration exceeding 240 min constitutes a notable risk factor for SSI (25). Similarly, Yang et al. demonstrated that extended operative time, specifically beyond 3 h, correlated with a twofold increase in SSI risk. Consistent with these findings, our study identified operative time as an independent risk factor for postoperative spinal infections. Through further investigation, we determined that when surgical duration surpassed 180 min, the postoperative infection rate was 7.7% (37 out of 483 cases). Regarding intraoperative blood loss, Liu et al. identified it as an independent risk factor for SSI (*p* = 0.005) (8). Our findings further reveal a significantly elevated postoperative infection rate of 7.6% (32 out of 422 cases) in patients experiencing intraoperative blood loss exceeding 475 mL. Salazar found that elevated blood loss during surgical procedures resulted in a decline in target hemoglobin levels, thereby diminishing the oxygen-carrying capacity and delivery, which consequently increased the risk of tissue necrosis (26). Furthermore, it has been proposed that the heightened incidence of wound infection following blood transfusion may be associated with altered pathology and immune response. However, this transfusion-related immunomodulatory mechanism has yet to be comprehensively validated through experimental studies (27).

In this study, we identified the suture method as a novel independent risk factor for postoperative spinal infection. This research is the first to report this association. Maria’s findings indicate that continuous intradermal running suture (IRS) is associated with reduced scarring and a lower incidence of postoperative incisional infections compared to simple interrupted percutaneous suture (SIPS) (28). Additionally, an experimental animal study demonstrated that the wound tensile strength in IRS-treated wounds was significantly greater, thereby accelerating the healing process relative to SIPS-treated wounds (29). Our retrospective analysis corroborates these findings, revealing a statistically significant reduction in postoperative infection rates with IRS (*p* < 0.05). Although our study is the first to identify suture method as an independent predictor of SSI, this finding should be considered exploratory. The association may be influenced by unmeasured confounders such as surgeon skill level, wound tension, or subcutaneous tissue management. Further prospective studies are needed to validate this relationship before clinical recommendations can be made.

The relationship between the surgical site and SSI has been highly debated. Cizik et al. reported that surgical site was closely related with SSI (30). Thoracic spine surgery has a higher reported incidence of SSI than lumbar or cervical procedures, likely attributable to its greater technical complexity, elevated risk, and prolonged operating time (19, 31). In our study, through multivariate regression analysis, the OR for thoracic compared with cervical was 0.412, suggesting a lower risk of infection. This may be related to the sample size distribution, differences in surgical techniques, or perioperative management at the participating centers, and further studies are needed to clarify this relationship. In this study, although we included the surgical site factor in the multivariate regression model, the final results showed that it was not an independent risk factor for SSI. Consistent with our findings, Han et al. have demonstrated the similar outcome (32). It was reported there was no significant correlation between surgical site (cervical, thoracic, and lumbar) and SSI. While surgical site was not an independent risk factor in our analysis, this does not entirely exclude its clinical relevance. The lack of significance may be due to the relatively small number of thoracic and cervical infections in our cohort or to the homogeneous surgical techniques across sites. Larger studies with balanced subgroup sizes are warranted to clarify this relationship.

The predominant organism isolated from the wounds of patients experiencing postoperative incisional infections was *Staphylococcus aureus* (Figure 7). Our findings align with conclusions from prior research (33, 34). In managing patients with incisional infections, we customarily administer vancomycin as an anti-infective agent prior to obtaining bacterial culture results. Although the prophylactic use of vancomycin is advocated in several international guidelines, a unified consensus has yet to be established (18, 35, 36). Some guidelines propose that the application of topical intra-wound vancomycin powder may reduce the incidence of SSIs; however, consensus is lacking due to insufficient evidence (37, 38). Patients with infections underwent surgical debridement and received intravenous antibiotics, and no cases of reinfection were observed following treatment.

In recent years, nomograms have been frequently employed to develop clinical models aimed at predicting the prognosis and risk of occurrence of associated diseases (39, 40). Nomograms derived from multifactorial analysis results facilitate individualized and precise predictions regarding the likelihood of medical events. These models enable the assessment of risks for each patient, can be effectively integrated into clinical practice, and enhance doctor–patient communication by forecasting patient outcomes (41). The AUC value of our nomogram was 0.787 (95% CI, 0.718–0.856), indicating that the calibration of the nomogram was relatively accurate. Consequently, this nomogram demonstrates a relatively high capability in predicting the SSI risk in patients.

## 5 Limitations

While the nomogram model exhibited high accuracy in predicting SSIs, several limitations were identified in this study. The use of retrospective data introduces the potential for selection bias. Furthermore, the study cohort consisted exclusively of Asian patients, which may limit the generalizability of the predictive results to individuals of other racial backgrounds. The retrospective nature of the study precluded the possibility of external validation, potentially introducing bias. Additionally, the absence of external validation and the retrospective design may overestimate model performance. The C-index of 0.787, while acceptable, leaves room for improvement, and clinical utility should be confirmed in prospective settings before routine application. Consequently, future research should involve validation with independent datasets and pursue large-scale, multicenter prospective studies to enhance the robustness and applicability of the findings.

## 6 Conclusion

In this study, we identified age, diabetes mellitus, albumin levels, operative time, suture method, and blood loss as independent factors associated with SSI. Based on these factors, the established nomogram model facilitates individualized treatment and early intervention for SSI. Future prospective external validation is warranted to further confirm the model’s generalizability.

## Data Availability

The original contributions presented in the study are included in the article/Supplementary material, further inquiries can be directed to the corresponding authors.

## Footnotes

1
http://www.r-project.org/

## Supplementary material

Supplementary Figure S1 The receiver operating characteristic (ROC) curve for the age.

Supplementary Figure S2 The receiver operating characteristic (ROC) curve for the albumin.

Supplementary Figure S3 The receiver operating characteristic (ROC) curve for the operative time.

Supplementary Figure S4 The receiver operating characteristic (ROC) curve for the blood loss.
