# Supplementary figures and images for: Nomogram for the prediction of surgical site infection following spinal surgery: a multicenter retrospective study
Source: Front Med (Lausanne). 2026 Apr 28;13:1832891. doi: 10.3389/fmed.2026.1832891 (PMC13160681; doi:10.3389/fmed.2026.1832891)

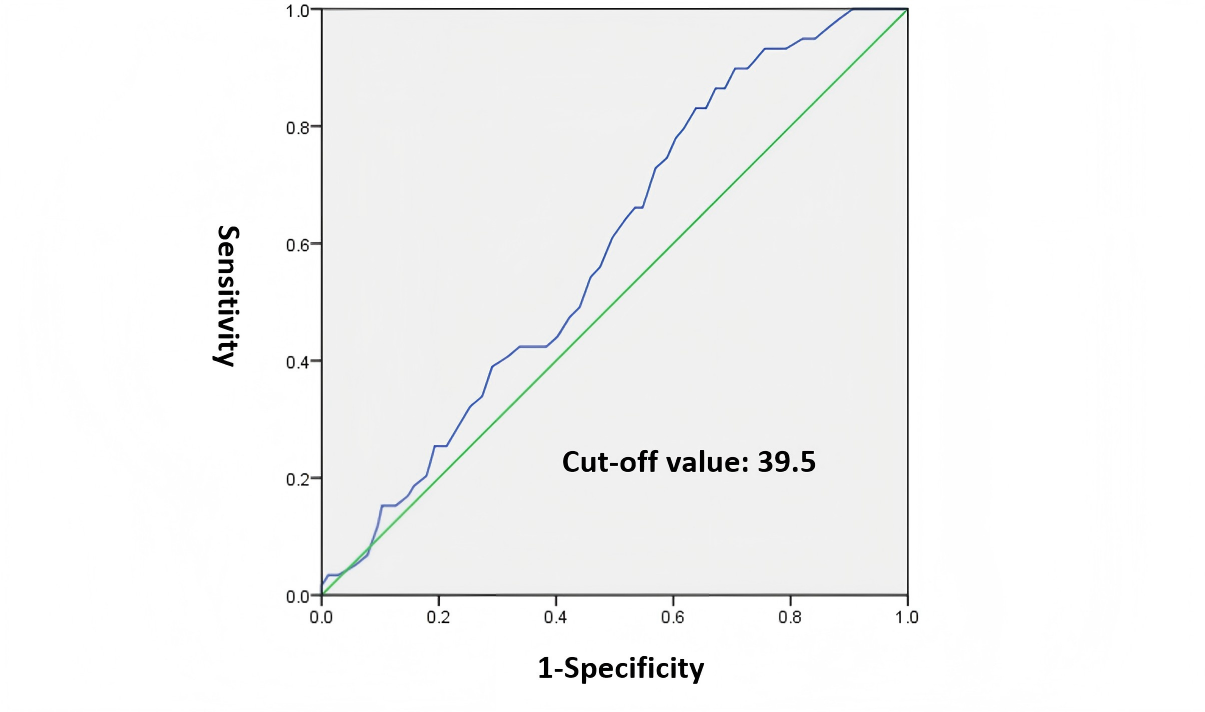

Supplement: Supplementary Figure S1 — The receiver operating characteristic (ROC) curve for the age. [file Image_1.tif]

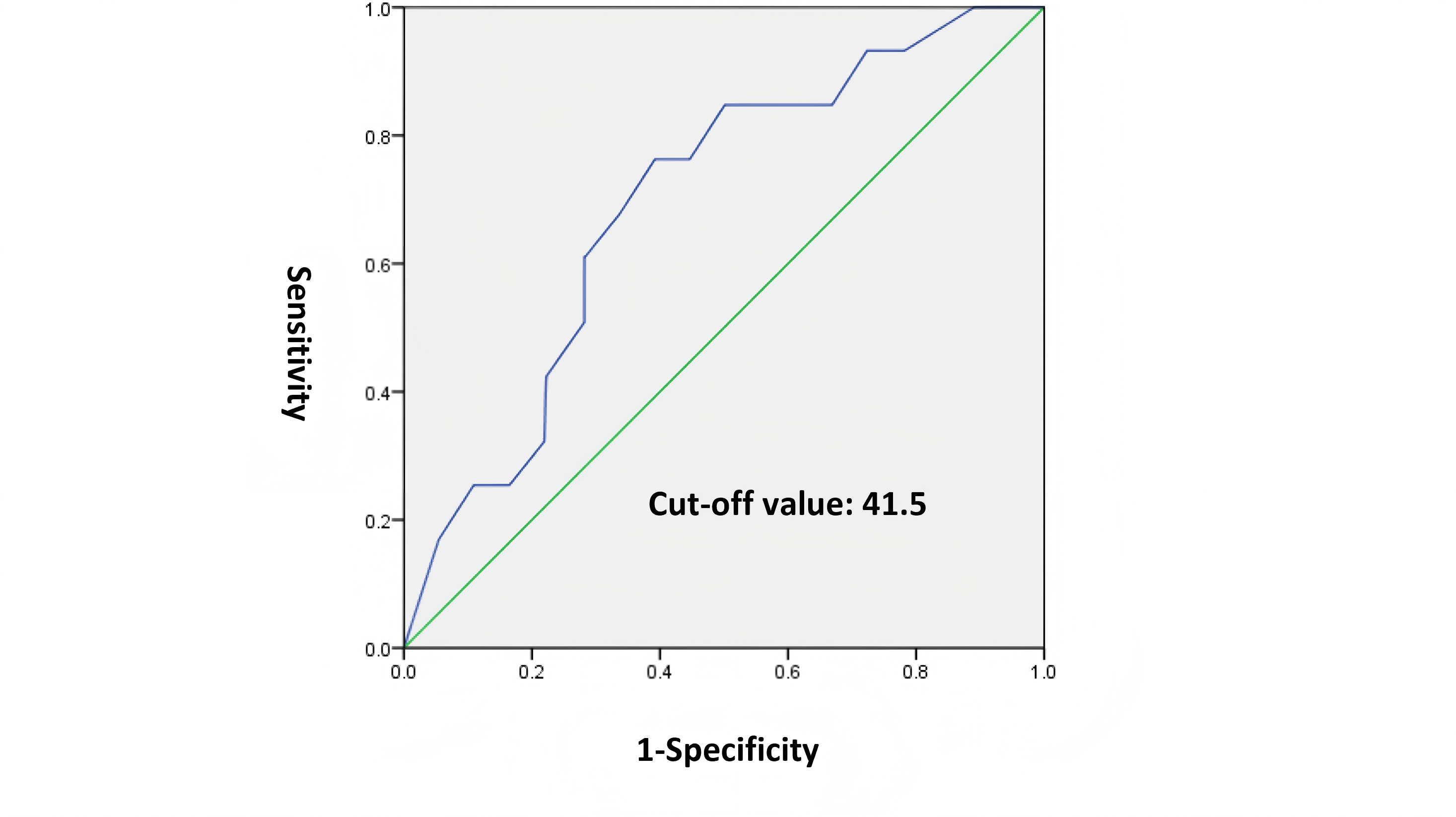

Supplement: Supplementary Figure S2 — The receiver operating characteristic (ROC) curve for the albumin. [file Image_2.tif]

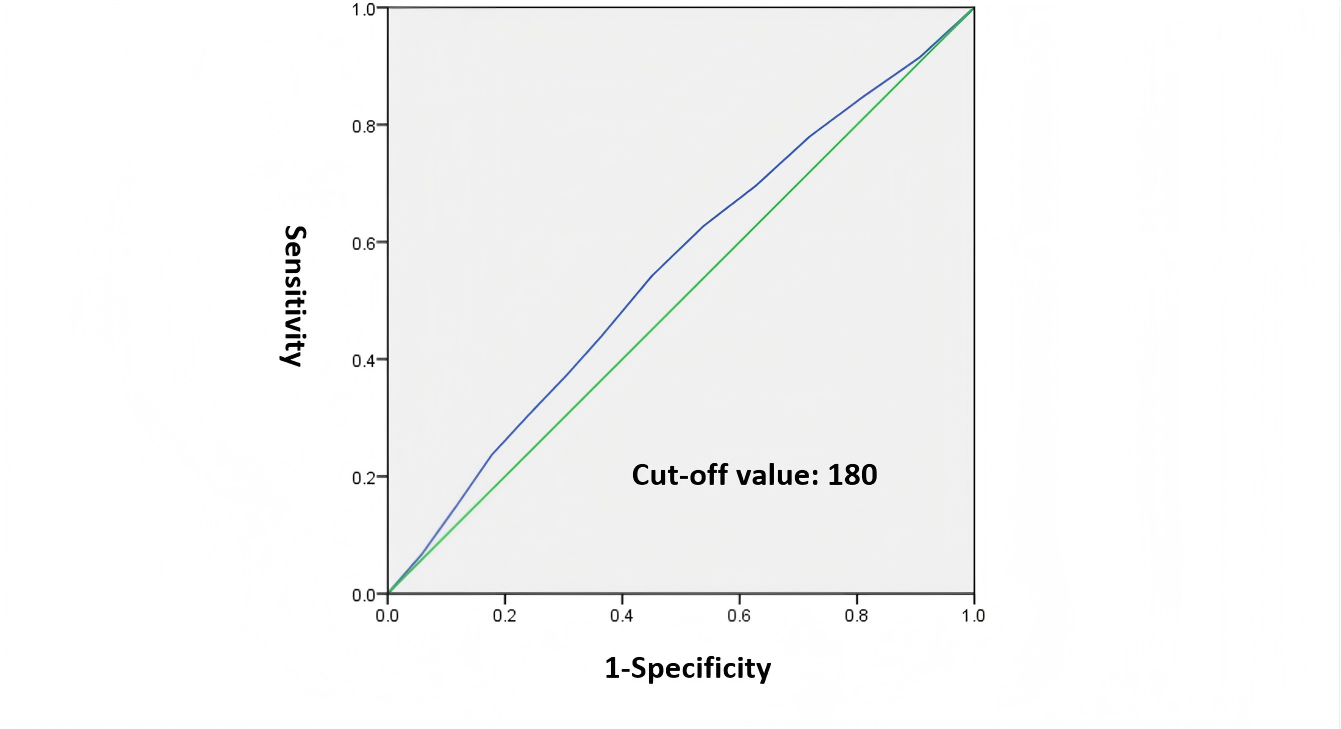

Supplement: Supplementary Figure S3 — The receiver operating characteristic (ROC) curve for the operative time. [file Image_3.tif]

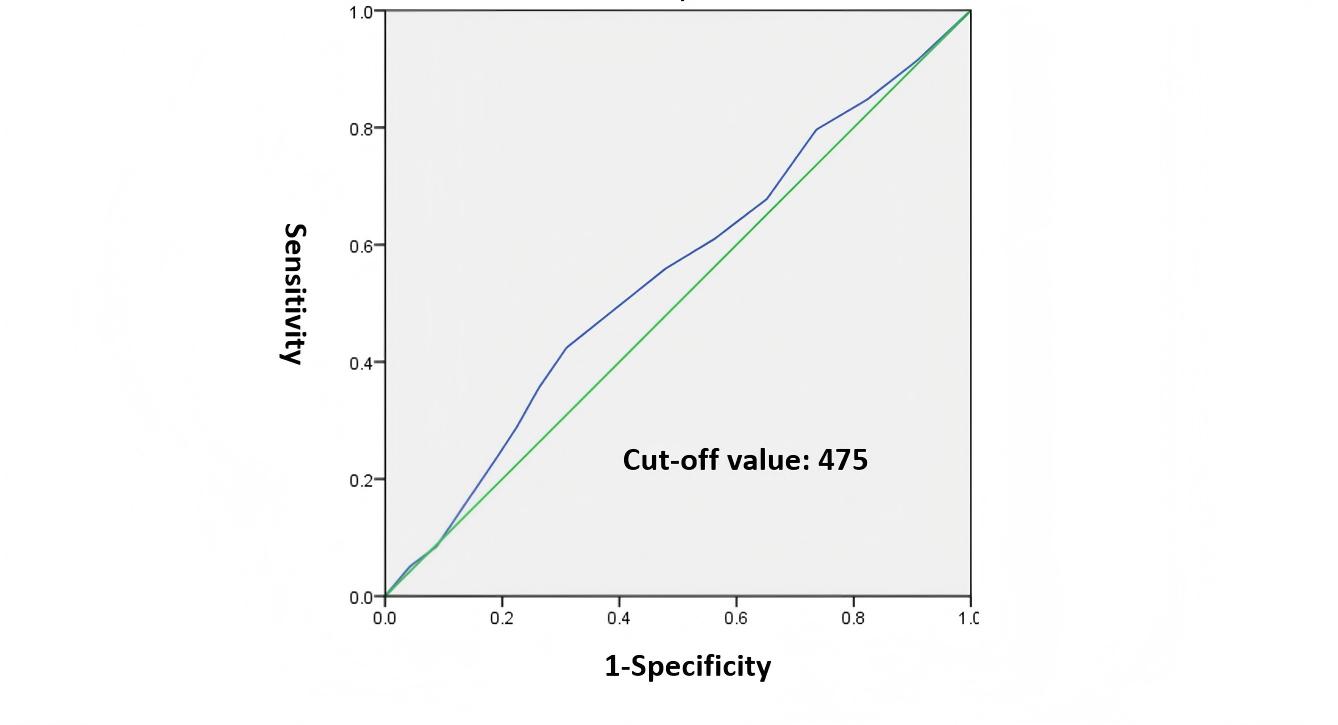

Supplement: Supplementary Figure S4 — The receiver operating characteristic (ROC) curve for the blood loss. [file Image_4.tif]
