# Supplementary material for: Nomogram for the prediction of surgical site infection following spinal surgery: a multicenter retrospective study
Source: Front Med (Lausanne). 2026 Apr 28;13:1832891. doi: 10.3389/fmed.2026.1832891 (PMC13160681; doi:10.3389/fmed.2026.1832891)
Supplement: Supplementary file 5 [file Table_1.docx]

**Table S1 Population Study Characteristics**

| **Characteristics** | **Value** |
| --- | --- |
| Male/Female, n (%) | 575(45.3%)/474(54.7%) |
| Age (years) | 56.03 ± 15.60 |
| Hypertension, n (%) | 522 (49.8%) |
| Cardiac disease, n (%) | 110 (10.5%) |
| Diabetes mellitus, n (%) | 117 (11.2%) |
| Active smoker, n (%) | 422 (40.2%) |
| Frequent alcohol consumption, n (%) | 313 (29.8%) |
| [Postoperative complication](javascript:;) n (%) |  |
| Rebleeding | 12 (1.14%) |
| Surgical site infection | 49 (4.67%) |
| New back pain | 13 (1.24%) |
| New leg pain | 15 (1.43%) |
| Persistent stenosis | 6 (0.57%) |
| Wound healing disorder | 13 (1.24%) |
| New dysesthesia | 16 (1.53%) |
| CSF leakage | 9 (0.86%) |
| Screw mispositioning | 10 (0.95%) |
| Follow-up (years) | 4.91 ± 1.16 |
| Data presented as mean ± standard deviation or n (%) | |
